# Data on heavy metals content and biochar toxicity in a pristine tropical agricultural soil

**DOI:** 10.1016/j.dib.2018.03.123

**Published:** 2018-03-31

**Authors:** Ihuoma N. Anyanwu, Moses N. Alo, Amos M. Onyekwere, John D. Crosse, Okoro Nworie, Clementina U. Uwa, Md. Faruque Hossain

**Affiliations:** a Lancaster Environment Centre, Lancaster University, Lancaster LA1 4YQ, United Kingdom; b Department of Biological Sciences, Federal University Ndufu-Alike Ikwo, PMB 1010, Ebonyi State, Nigeria; c American International University-Bangladesh, Dhaka 1213, Bangladesh

## Abstract

Accumulation of heavy metals results in soil degradation and impairs the normal functioning of ecosystems. Thus, monitoring of heavy metals is essential in both pristine and polluted soils. Concentrations of heavy metals were determined in a pristine tropical agricultural soil using acid digestion procedures. The soil samples were also analyzed for physico-chemical parameters and biochar toxicity to earthworms. Data shows that the soil is acidic, with low organic matter content. The level of heavy metals ranged from <0.06±0.0 to 595.8±2.8 µg g^−1^. However, the concentrations were found to be below the soil regulatory standards of heavy metals in agricultural soils. Furthermore, increased addition of biochar to the soil caused toxic effect on earthworms over a 90 d biochar-soil contact time. The data provides baseline information of heavy metals in pristine agricultural soils from the region, and the effect of biochar amendments on tropical soils.

**Specifications Table**

**Table:** 

Subject area	Environmental Science
More specific subject area	Soil Science, Chemistry, Ecotoxicology
Type of data	Table, Figure
How data was acquired	Atomic Absorption Spectroscopy (PerkinElmer AAnalyst 200), Earthworm toxicity test.
Data format	Analyzed, raw
Experimental factors	Prior analysis, soil was sieved with 2 mm mesh size, amended with biochar.
Experimental features	Analysis of soil physico-chemical parameters, heavy metals, biochar toxicity test
Data source location	Ebonyi State, South-East, Nigeria
Data accessibility	Data available in the article
Related research article	Anyanwu et al. (2018) [1].

**Value of the Data**

• Data shows the physico-chemical properties of a tropical agricultural soil.
• Data provides baseline information on the level of heavy metals in a pristine agricultural soil from the region.
• Data would help in effective remediation of heavy metals contaminated soils in the area.
• Data portrays the effect and bioavailability of biochar to earthworms in soil over time.

## 1 Data

Data provided in this brief were generated during soil preparation/analysis of the experiment on ‘influence of biochar aged in acid soil on ecosystem engineers and two tropical agricultural plants’. These data supplement the investigation on physico-chemical properties of the test soil. Also, the impact of the amended biochar was measured on earthworms’ survival over a 90 d biochar-soil contact time.

### 1.1 Physico-chemical properties

The physico-chemical compositions of the soil are shown in Table 1. From the data, the soil texture can be classified as clay loam, with low organic matter content. The soil is also very acidic with a pH of 5.4. Having high percentage of clay, this type of soil (soil texture) be hard when dry and sticky when wet. It can have very high water retaining capacity and poor permeability.

**Table 1:** Physico-chemical properties and heavy metals content of a pristine tropical agricultural soil.

**Analyte/Properties**	**Unit**	**Parameter value**
Soil texture	% dry weight	Clay-loam
Organic matter content	% dry weight	14.5±0.0
pH	pH unit	5.4±0.0
Manganese (Mn)	µg g^−1^	22.6±1.7
Lead (Pb)	µg g^−1^	16.6±3.4
Copper (Cu)	µg g^−1^	7.5±1.3
Iron (Fe)	µg g^−1^	595.8±2.8
Cadmium (Cd)	µg g^−1^	<0.06±0.0
Zinc (Zn)	µg g^−1^	7.87±0.1
Calcium (Ca)	µg g^−1^	0.21±0.2

Data shows mean±standard error.

### 1.2 Concentrations of heavy metals

Heavy metals are ubiquitously distributed in soil and monitoring of metals is important to identify contaminated agricultural soils. The levels of selected heavy metals (Manganese (Mn), Lead (Pb), Copper (Cu), Iron (Fe), Cadmium (Cd), Zinc (Zn), Calcium (Ca)) were determined in a pristine tropical agricultural soil. The individual heavy metals measured from the soil samples ranged from <0.06±0.0 to 595.8±2.8 µg g^−1^ (Table 1). The mean value of the analytes followed the descending order: Fe>Mn>Pb>Zn>Cu>Ca>Cd. Some heavy metals (Cu, Fe, Mn, Zn) are considered essential for living organisms, but can be toxic at high concentrations. These metals occur naturally, but soil concentrations can be elevated by anthropogenic process. In this pristine agricultural soil, Iron (Fe) measured the highest concentration of 595.8±2.8 µg g^−1^. Also, Cadmium (Cd) and Lead (Pb) in soil may be associated with human activities and both of these elements have no beneficial biological function. Cd recorded the lowest value of <0.06±0.0 µg g^−1^ while Pb, measured 16.6±3.4 µg g^−1^. However, the concentrations of heavy metals in the pristine agricultural soil measured below the regulatory standards (of heavy metals) in agricultural soils [6], [7], [8], [9]. Soil metal concentrations in the pristine agricultural soil from this study were compared with other heavy metals data on agricultural soils carried out in the Spanish region (Table 2). The soil heavy metal concentrations were found lower than the concentrations determined by other researchers on Spanish and Iran agricultural soils [10], [11], [12], [13].

**Table 2:** Data comparison with others on agricultural soils from Spanish region (mg kg^−1^).

**Analyte/Properties**	**This data**	**Mico et al.**[11]	**Campos**[10]
Soil texture	Clay-loam	Silty clay loam	–
Organic matter content	14.5	2.2	–
pH	5.4	8.2	>7
Manganese (Mn)	22.6	320	533
Lead (Pb)	16.6	19.6	64
Copper (Cu)	7.5	21.6	20
Iron (Fe)	595.8	15,274	34,000
Cadmium (Cd)	<0.06	0.38	2.3
Zinc (Zn)	7.9	57.8	90
Calcium (Ca)	0.21	–	–

### 1.3 Earthworm toxicity test

**Fig. 1:**
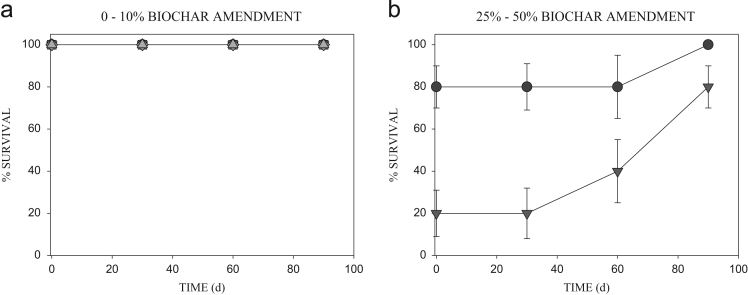
Survival (%) of *E. eugeniae* exposed to soils amended with rice husk biochar and aged for different periods of time. Data shows: 0% (•), 0.5% (▼), 1% (■), 5% (◆), 10% (▴) (a), and 25% (•), 50% (▼) (b), *n*=10. Maximum standard of error=±20.41.

From the data, 0% earthworm mortality was recorded in soils with 0.5–10% biochar addition, over the 90 d soil contact time. Nevertheless, increase in biochar addition resulted in increased mortality of the earthworms (*p*<0.05; Fig. 1). Of the amendments, 50% biochar was the most toxic to the exposed earthworms during the 90 d biochar-soil contact time. The graph showed decline in toxicity with increase in biochar-soil contact time. Furthermore, soils amended with 25% biochar recorded 100% survival of earthworms after 90 d; while, 80% survival was recorded in the 50% soils (Fig. 1b). The soil pH (5.40±0.0), type of char and/or char bioavailability, may have probably contribute to the observed toxicity. The reduced toxicity recorded over time may be as a result of biochar sorption or biodegradation.

## 2 Experimental design, materials, and methods

### 2.1 Physico-chemical properties

Prior analysis, the soil samples were air dried and sieved with 2 mm mesh size. Soil texture was determined using manipulative texturing (by hand). Soil pH was measured with pH radiometer model PHM 93 (Copenhagen) and the organic matter content was determined by loss on ignition (after baking in the furnace for 16 h at 450 °C).

### 2.2 Acid digestion procedures

Three 10 g of soil sample were digested in 20 ml of concentrated nitric acid (Primar Plus, Fisher Scientific) for 2 h under reflux conditions. After digestion, excess acid was carefully boiled off and re-dissolved with 10 ml 5% nitric acid. Blank determinations (acid only) were run at the same time with samples. The extracts were then filtered under gravity using Whatman 542 filter paper and made up to 25 ml with MilliQ water in volumetric flasks containing 1 ml of 2% w/v lanthanum chloride (AAS grade, Fisher Scientific).

Prior to use, all glassware were soaked for 24 h in 10% nitric acid (Primar Plus, Fisher Scientific) and rinsed with deionized water. The deionized water was MilliQ 18.2 MΩ; organic carbon <2 ppm.

### 2.3 Sample analysis

Each extract was analyzed in triplicate (relative standard deviation <1%). The instrument was calibrated using a 5 point calibration from zero up to 20 mg kg^−1^ (0–20 mg kg^−1^) depending on the analyte using AAS grade standards (Fisher Scientific). Three procedural blanks were run in parallel and sample concentrations were blank subtracted. The instrument response (signal to noise) was 2:1. The instrument LoD was 0.05–0.5 ppm and a sample LoD ranged from 0.02 to 0.2 mg kg^−1^.

### 2.4 Earthworm toxicity test

Toxicity of biochar to earthworms in soil was determined using the guidelines described in OECD 207 and 222 [4], [5]. Mature earthworms (*Eudrilus eugeniae*) weighing 0.5–0.7 g were used. Before the 14 d exposures, earthworms were washed and left in Petri dishes with moist filter paper to void their gut content for 24 h, after which the earthworms were weighed [1], [2], [3]. Triplicates of 10 earthworms were used for each amendment (0%, 0.5%, 1%, 10%, 25% and 50% dry biochar kg^−1^ soil). Soils (600 g) were weighed into glass trays, deionized water was added to moisten the soil to 80% WHC; mixed thoroughly and 10 earthworms were added to each replicate (without food). The samples were covered with perforated foil and incubated in the dark at 24±2 °C. Mortality was determined after 14 d of exposure [1], [2], [3].

## References

[1] Anyanwu I.N., Alo M.N., Onyekwere A.M., Crosse J.D., Nworie O., Chamba E.B. (2018). Influence of biochar aged in acidic soil on ecosystem engineers and two tropical agricultural plants. Ecotoxicol. Environ. Saf..

[2] Anyanwu I.N., Clifford O.I., Semple K.T. (2017). Effects of single, binary and quinary mixture of phenanthrene and its N-PAHs on Eisenia fetida in soil. Water Air Soil Pollut..

[3] Anyanwu I.N., Semple K.T. (2016). Effects of phenanthrene and its nitrogen-containing analogues aged in soil on earthworm Eisenia fetida. Appl. Soil Ecol..

[4] OECD (1984). Earthworm, Acute Toxicity Tests. OECD Guidelines for Testing of Chemicals, Section 2, Effects on Biotic Systems.

[5] OECD (2016). Earthworm Reproduction Test (Eisenia fetida/ Eisenia andrei). OECD Guideline for the Testing of Chemicals.

[6] United States Environmental Protection Agency (2002). Supplemental Guidance for Developing Soil Screening Levels for Superfund Sites. http://www.epa.gov/superfund/health/conmedia/soil/index.htm.

[7] European Environmental Agency. Progress in management of contaminated sites (CSI 015/LSI 003), 〈http://www.eea.europa.eu/data-and-maps/indicators〉, 2007.

[8] Canadian Ministry of the Environment. Soil, Ground Water and Sediment Standards for Use Under Part XV.1 of the Environmental Protection Act, 2009.

[9] Environmental Protection Ministry of China. Standards of Soil Environmental Quality of Agricultural Land. Huangbanhang 69. Office of Environmental Protection Ministry of China, Beijing, China, 2015.

[10] Campos E. (1997). Estudio de la contaminación y fraccionamiento químico de metales pesados en suelos de la Vega de Granada (Doctoral Thesis).

[11] Micó C., Peris M., Sánchez J., Recatalá L. (2006). Heavy metal content of agricultural soils in a Mediterranean semiarid area: the Segura River Valley (Alicante, Spain). Span. J. Agric. Res..

[12] Kamani H., Mahvi A.H., Seyedsalehi M., Jaafari J., Hoseini M., Safari G.H., Dalvand A., Aslani H., Mirzaei N., Ashrafi S.D. (2017). Contamination and ecological risk assessment of heavy metals in street dust of Tehran Iran. Int. J. Environ. Sci. Technol..

[13] Naghipour D., Jaafari J., Ashrafi S.D., Mahvi A.H. (2017). Remediation of heavy metals contaminated silty clay loam soil by column extraction with ethylenediaminetetraacetic acid and nitrilo triacetic acid. J. Environ. Eng..

