# Supplementary material for: Data on heavy metals content and biochar toxicity in a pristine tropical agricultural soil
Source: Data Brief. 2018 Mar 31;18:1064–8. doi: 10.1016/j.dib.2018.03.123 (PMC5996615; doi:10.1016/j.dib.2018.03.123)
Supplement: Supplementary file 1 — Supplementary material [file mmc1.docx]

I declare that the authors whose names are listed in the paper have No conflict of interest.
